# Genetic analysis of NS5B gene from bovine viral diarrhea virus-infected cattle in Central and East Java, Indonesia

**DOI:** 10.14202/vetworld.2019.1108-1115

**Published:** 2019-07-25

**Authors:** S. H. Irianingsih, H. Wuryastuty, R. Wasito, H. Wibawa, F. S. Tjatur Rasa, B. Poermadjaja

**Affiliations:** 1 Doctoral Study Program, Faculty of Veterinary Medicine, Universitas Gadjah Mada, Yogyakarta, Indonesia; 2 Disease Investigation Centre Wates, Yogyakarta, Indonesia; 3 Department of Veterinary Internal Medicine, Faculty of Veterinary Medicine, Universitas Gadjah Mada, Yogyakarta, Indonesia; 4 Department of Veterinary Pathology, Faculty of Veterinary Medicine, Universitas Gadjah Mada, Yogyakarta, Indonesia; 5 Directorate of Animal Health, Directorate General of Livestock Services and Animal Health, Ministry of Agriculture, The Republic of Indonesia, Jakarta, Indonesia

**Keywords:** bovine viral diarrhea virus, NS5B gene, phylogenetic analysis, point mutation, subgenotype

## Abstract

**Background and Aim:**

A previous study divided Indonesian bovine viral diarrhea virus (BVDV)-1 into subgenotypes BVDV-1a to BVDV-1d based on the partial NS5B gene using strain Bega as reference for BVDV-1a. In fact, it is clustered into BVDV-1c with strain Bega-like Australia. BVDV genotyping has been done on isolates from Jakarta, West and Central Java, but East Java isolates have not been genotyped. This study aimed to analyze genetic variability and amino acid residues in the nucleotide-binding pocket of the NS5B gene from infected cattle.

**Materials and Methods:**

Samples were obtained from the Sera Bank originating from active and passive surveillance of cattle that had been tested for BVDV antigen from 2013 to 2017. Detection of the p80 antibody and BVDV genotyping was carried out using ELISA and nested-multiplex-polymerase chain reaction (PCR), respectively. We defined 15 nested PCR products for partial sequencing of NS5B. Those field samples were selected from each location and year using proportional calculation as a representative sample. Homological and phylogenetic analyses of the partial NS5B gene were performed using BLAST and MEGA version 6.

**Results:**

Based on the phylogenetic tree analysis using 360 nucleotides as the partial NS5B gene, Indonesian BVDV-1 isolates from Central and East Java were subdivided to BVDV-1a (n=9), BVDV-1b (n=1), and BVDV-1c (n=5). In the present study, the homology of BVDV subgenotype -1a, -1b, and -1c was compared to the BVDV GenBank data and found 90-93%, 93%, and 92-95% respectively with the average pairwise distance of 0.207. A point mutation was shown at R283K of all BVDV isolates based on the sequence of three amino acid residues R283, R285, and I287 in the nucleotide-binding pocket as a part of the encoded RNA-dependent RNA polymerase.

**Conclusion:**

This study revealed the genetic variability of BVDV infecting cattle in Central Java and East Java, Indonesia, the subtypes BVDV-1a, BVDV-1b, BVDV-1c, and a point mutation at the R283K residue.

## Introduction

Bovine viral diarrhea virus (BVDV) is an important viral pathogen of cattle that has spread globally and that causes significant economic loss to both dairy and beef cattle [1]. BVDV causes thousands and up to tens of millions of dollars of loss per calving interval [2] due to productivity and reproductive disorders in the herd [3]. Around 70-90% of infected cattle show no clinical signs [4-6]. The immunosuppressive condition may increase both the risk of secondary infection and inefficient reproduction and productivity. The BVDV genome is a single-stranded positive-sense ribonucleic acid (RNA) belonging to the genus *Pestivirus* and the family Flaviviridae [7]. The BVDV genome is about 12.3 kb long, which organized as an open reading frame flanked by 5’- and 3’-untranslated regions (UTR) [8-10]. It encodes a single polyprotein of about 4000 amino acids consisting of proteins in the order of NH2-Npro-C-Erns-E1-E2-P7-NS2-NS3-NS4A-NS4B-NS5A-NS5B-COOH. The BVDV can be categorized into two genotypes or species: BVDV-1 and BVDV-2 [11]. Based on the nucleotide sequence variation in the 5’ UTR [12] and four other regions including Npro, E2, NS3, and NS5B–3’UTR [13], the genotypes BVDV-1 and BVDV-2 can be divided into numerous subgenotypes. Nonstructural NS5B was classified as a highly conserved gene [14] with a nucleotide length of 2.156 bp [15]. This specific gene can be used to determine the genotype using nested multiplex polymerase chain reaction (PCR) [16], which can be used for phylogenetic analysis and further characterization [13]. This gene encodes RNA-dependent RNA polymerase (RdRP), which is responsible for replication and transcription of the viral genome [17] through proofreading [18]. Its activity could be predicted through the F-motif sequence in RdRP consisting of the conserved residues R283, R285, and I287 [19].

In Indonesia, the first BVD case was reported in Bali cattle in 1989 in Sulawesi [20], and there were no other reports until 2009. About 43.2% of beef, dairy, and breeding cattle were seropositive for BVDV [21]. The prevalence tended to increase up to 46% by 2013 [22,23]. According to another study, the high BVDV seroprevalence caused significant damage to the production industry [24]. The previous BVDV genotyping study of diarrhea and respiratory disorders in cattle detected subgenotypes -1a to -1d based on the NS5B gene with -1a predominantly in Indonesia [25]. However, the phylogenetic tree is not precise and robust, because the reference BVDV-1 strain Bega should cluster as BVDV-1c with strain Bega-like instead of BVDV-1a. BVDV genotyping has been done in isolates from Jakarta, West Java and Central Java, but there is no information from East Java BVDV isolates. BVDV disease control requires updated data and information about genetic variability to design and construct a vaccine for future.

The phylogenetic analysis was used to determine the infection source, genetic variability in virus generation, and the pestivirus classification. The determination of the pestivirus subgroup and the development of guidelines for investigation and classification are very important in epidemiology studies [26]. This study used the serum as samples, the district as location, and NS5B as the basis of genotyping to improve and update the previous study’s phylogenetic analysis. These findings suggested that BVDV genetic variability determination using the partial NS5B gene is useful for controlling and eradicating BVDV. Hopefully, these methods may be used in areas with active BVDV infections.

This study aimed to analyze the genetic variability of the NS5B nucleotide sequence in BVDV isolated from infected cattle in Central Java and East Java, Indonesia, between 2013 and 2017 and to analyze the amino acids residues in the motif F sequence of the RdRP enzyme.

## Materials and Methods

### Ethical approval

This study did not work on human or animal, only worked on serum sample obtained from diagnostic laboratory, so, it does not need ethical approval.

### Sera collection

A total of 61 BVDV-positive serum samples from the Sera Bank at the Disease Investigation Centre (DIC), Wates were used in this study. The sera were collected from active and passive surveillance in cattle by DIC Wates between 2013 and 2017. A total of 9845 sera samples were initially analyzed using BVDV antigen-capture ELISA (ACE). Sera that had good quality and quantity and gave a positive result for the BVDV antigen using ACE were collected as sera bank and kept at −80°C until analysis.

### Serological test

The Ag-capture ELISA test of BVDV was carried out using the BVDV ELISA antigen kit (Idexx^®^) to detect antigen Erns of the BVDV. The test was considered as a screening test for persistently infected cattle. The BVDV antibody was analyzed using IDVet ID Screen^®^ BVD p80 Antibody Competition kit.

### RNA isolation and nested-multiplex PCR

The viral RNA from sera samples was extracted using the Viral Nucleic Acid Kit II (Geneaid^®^, Geneaid Biotech Ltd., Taiwan) as described by the manufacturer. The extracted RNA was subjected to the nested-multiplex PCR BVD test followed by agarose gel resolution to genotype the BVDV.

The specific NS5B primers used for external reaction and nested-multiplex genotyping are listed as Table-1 [16]. The first step external reverse transcriptase reactions were performed using the AffinityScript One-Step RT-PCR Kit (Agilent, Santa Clara, CA, USA) as specified by the manufacturer. The thermal conditions for the external reactions were as follows: 45°C for 30 min of reverse transcription, 92°C for 1 min of initial denaturing followed by 40 cycles of 92°C for 20 s, 50°C for 20 s, 68°C for 45 s, and final elongation at 68°C for 5 min. The second stage of the multiplex PCR genotyping was done using HotStar Taq Mastermix Kit (Qiagen^®^, Hilden, Germany). The reaction was heated to 95°C for 15 min, followed by 35 cycles of 94°C for 40 s, 56°C for 40 s, 72°C for 40 s, with a final elongation step of 72°C for 7 min.

**Table 1:** Primers used for nested-multiplex genotyping.

PCR step	Genotype	Primer sequence (5’- 3’)	Position	Product
First/external	BVDV	Fwd: AAGATCCACCCTTATGA (A/G) GC	10385-10404	1.100 bp
		Rev: AAGAAGCCATCATC (A/C) CCACA	11528-11547	
Second/mutiplex	BVDV-1 BVDV-2	Fwd-1: TGGAGATCTTTCACACAATAGC	10758-10779	360 bp 604 bp
		Fwd-2: GGGAACCTAAGAACTAAATC	10514-10533	
		Rev: GCTGTTTCACCCAGTT (A/G) TACAT	11096-11117	

Fwd=Forward, Rev=Reverse; reference[16]. PCR=Polymerase chain reaction

BVDV-1 strain Singer was used as a positive control for genotype-1 in this study. All the PCR products were separated on a 1.5% agarose gel, stained with SYBR^®^ Safe DNA gel stain, and visualized using Gel Documentation Systems from Bio-Rad.

### Sequencing and sequence analysis

Sequencing was conducted on the second step nested-multiplex PCR positive samples to confirm the BVDV genotype based on the partial NS5B gene. We selected 15 nested PCR products for sequencing from each location and year using proportional calculation as representative sample to eliminate biases. We considered the number of positive nested-multiplex PCR based on location and specified the year of collection. The selected PCR products were sequenced in the 1^st^ BASE, Malaysia. Sequence analysis of the partial NS5B gene was performed usingMEGA version 6 [27]. Alignment analysis of the nucleotide sequences of 15 samples from known BVDV strains retrieved from GenBank was performed using the MUSCLE algorithm. Phylogenetic trees were constructed using the maximum likelihood method with the Tamura Nei 93 substitution model, Gamma G+I distribution, and 1000 replication bootstraps in MEGA 6. The sequence identity of 15 samples and their protein translations into amino acids were analyzed usingBioEdit version 7.2.5 [28]. The analysis aimed to discover the genetic variability of BVDV and the residues in the motif F sequence of the RdRP enzyme coding sequence [19].

## Results

### Detection of p80 antibodies in BVDV antigen-identified serum samples

A total of 61 serum samples with BVDV antigens detected from 2013 to 2017 were used to detect antibodies against p80 BVDV. Twelve out of sixty-one sera contained BVDV p80 (NS2-3) antibodies (19.7%). The ratios of seropositive and seronegative BVDV p80 to BVDV antigen-positive from 2013 to 2017 were 2/11, 1/13, 6/22, 3/2, and 0/1, respectively.

### Nested-multiplex PCR

**Figure-1:**
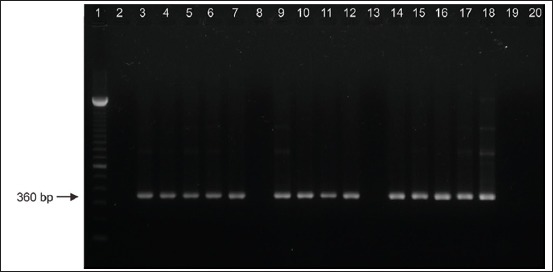
The nested-multiplex polymerase chain reaction of the NS5B gene for bovine viral diarrhea virus (BVDV)-1 genotyping using serum samples with the prediction size of 360 bp. (Lane 1: DNA marker 100 bp, lane 2-17: BVDV-1 positive samples, lane 18: BVDV-1 strain Singer positive control, lane 19: Negative control, lane 20: Non template control).

The nested-multiplex PCR detected and typed BVDV from serum samples based on a single band (Figure-1). Forty-six out of 61 samples showed the genotype BVDV-1 based on the BVDV-1 strain Singer as a positive control, and the PCR products were specific for BVDV type 1 at 360 bp. No fragments were obtained using primers for genotype BVDV-2 with a predicted size of 604 bp. Based on the serology test, the BVDV-1-positive serum samples were 80% (37/46) seronegative and 20% (9/46) seropositive against p80 BVDV from 2013 to 2017 as shown in Table-2.

**Table 2:** Comparison between BVDV p80 antibody and genotyping detection based on multiplex nested PCR.

Year	No. of samples	BVDV seropositive		BVDV seronegative	

		Nested-multiplex RT-PCR			

		BVDV-1 (+)	BVDV-1/-2 (-)	BVDV-1 (+)	BVDV-1/-2 (-)
2013	13	2	0	10	1
2014	14	1	0	9	4
2015	28	4	2	16	6
2016	5	2	1	2	0
2017	1	0	0	0	1
Total	61	9	3	37	12

RT-PCR=Reverse transcription-polymerase chain reaction, BVDV=Bovine viral diarrhea virus

### Nucleotide sequencing and phylogenetic tree analysis

All BVDV-1 positive samples detected originated from 4 districts in the Central Java province and 2 districts in the East Java province. The Central Java districts with positive samples were Banyumas (n=36), Cilacap (n=5), Semarang (n=2), and Boyolali (n=1). The two East Java districts were Pasuruan (n=1) and Malang (n=1). We considered positive field samples based on the year of collection from 2013 (n=5), 2014 (n=2), 2015 (n=6), and 2016 (n=2). We selected 15 BVDV-1-positive samples (around 30%) for sequencing based on the proportional ratio between each number of districts and the total as a representative location from 2013 to 2016. We selected 9 samples. Two samples were from Banyumas and Cilacap, and the 4 remaining samples came from Semarang, Boyolali, Pasuruan, and Malang.

**Figure-2:**
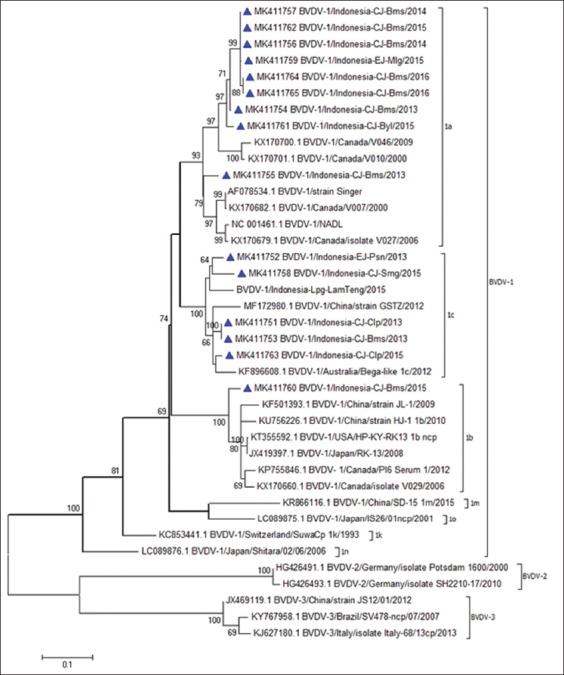
Phylogenetic tree illustrating the subgenotypes of bovine viral diarrhea virus samples based on partial NS5B gene (360 nt). The phylogenetic tree was constructed using the maximum likelihood method implemented in MEGA 6 with bootstrap values as 1000 replicates. The bar indicates the substitutions per site. Accession numbers are listed at the beginning of sample names.

Phylogenetic tree reconstruction for genotyping of the BVDV-1 field samples was done using the 360 nucleotide NS5B gene. Genetic analysis of the partial NS5B gene revealed that a total of three subgenotypes were present in the samples: BVDV-1a (60%), BVDV-1b (6.67%), and BVDV-1c (33.33%) as illustrated in Figure-2. The nucleotide sequence identity value was closer to the reference, nine BVDV-1a field samples to KX170701 (BVDV-1/Canada/V010/2000) 90-93%, BVDV-1b field sample to KP755846 (BVDV-1/Canada/PI6 serum 1/2012) 93%, and five BVDV-1c field samples to KF896608 (BVDV-1/Australia/Bega-like_1c/2012) 92-95%.

The mean distance within the group among BVDV-1a, BVDV-1b, and BVDV-1c was 0.071, 0.079, and 0.067, respectively. The mean distance between group BVDV-1a and BVDV-1b was 0.166. The mean distance between group BVDV-1a and BVDV-1c was 0.15. The mean distance between group BVDV-1b and BVDV-1c was 0.167. The average of all pairwise distances from 15 BVDV-1 field samples and 23 references retrieved from GenBank was 0.207.

### Alignment of predicted amino acid sequences

**Figure-3:**
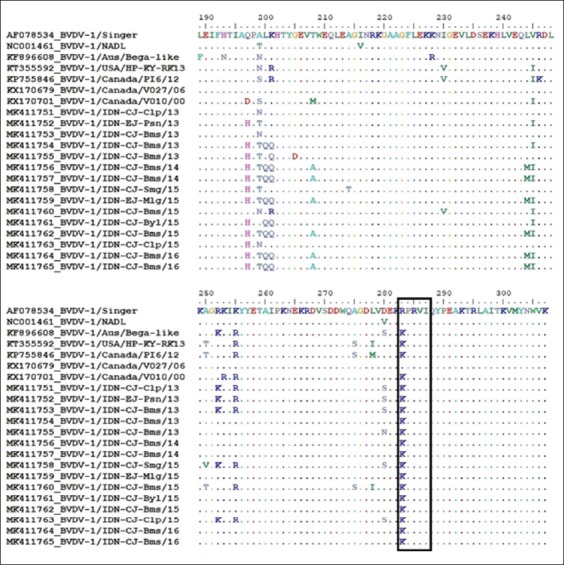
Alignment of consensus sequences of fifteen samples and predicted amino acid residues (189-307) from the NS5B gene of the circulating viral populations. The RNA-dependent RNA polymerase enzyme encodes amino acid that contained conserve residues of R283, R285 and I287 inside the box.

The analysis of the subsequence 189-308 in the 360 bp NS5B nucleotide sequence was conducted. The alignment of the entire 120 amino acid NS5B sequence for fifteen samples showed that residues R283K, R285, and I287 were in the nucleotide-binding pocket, as illustrated in Figure-3. The conserved residues K263, R267, K282, R283, R285, and I287, were in the F-motif sequence. Two sequences retrieved from GenBank, BVDV-1 strain Singer and NADL had no changes in R283, but all samples and other reference sequences had point mutations in the residue R283K.

## Discussion

Serum samples were tested for the presence of BVDV p80 antibody using commercially available Ab-ELISA. The presence of the p80 antibody in the samples might be caused by either horizontally transmitted acute infection or infected pregnant cows. Based on the survey report from DIC Wates, the farms did not have any vaccination history. Therefore, the 19.7% seropositive rate did not result from the vaccination cattle [22]. The BVDV p80 antibody is an NS3 protein from natural exposure [29], and it could be detected within 2 to 3 weeks after infection [19]. In Central Java, 56.2% of dairy cattle that were not vaccinated with BVDV and had a history of reproductive disorders showing antibodies against the nonstructural protein NS2-3 BVDV. This showed that dairy cattle had been exposed to circulated BVDV rather than vaccination [23].

Samples with both seropositive, ACE and nucleic acid detection might indicate an acute infection, and ACE and nucleic acid positive, but seronegative might suggest either an early phase of acute infection or a persistent infection [30]. Aside from being a source of transmission of the virus, PI is important because it also indicates viral variability and represents a unique evolutionary model [31]. It should be considered for further sequencing analysis. These 46 samples showed positive BVDV antigen, and they were comprised of 37 seronegative samples and 9 seropositive samples. The samples might be from acute infection and persistent infection cattle in the fields, but PI cattle serve as lifelong reservoirs [32].

The nested-multiplex PCR of the NS5B gene could differentiate between the BVDV-1 and BVDV-2 genotypes for initial screening of the BVDV-infected samples. Here, BVDV-1 was detected using nested-multiplex PCR in 46 out of 61 (75%) field samples from Central Java and East Java between 2013 and 2017, but BVDV-2 was not detected from all samples tested. Based on previous studies in Indonesia, the reported BVDV genotype was BVDV-1 [25,33,34]. Based on the BVDV nucleotide sequences that were published, there are considerably more virus isolates described as BVDV-1 (88.2%) than BVDV-2 (11.8%) [35].

Further genetic analysis using 15 BVDV-1 field samples based on location and the year of sample collection was presented. Based on the serology test, samples for sequencing consist of 12 p80 antibody seronegative and 3 seropositive BVDV samples. The alignment between NS5B nucleotide sequences from samples and 23 GenBank references were reconstructed in a phylogenetic tree. In this study, the phylogenetic tree is more robust than a previous study which had no information about bootstrap replication, branch scores, or accession numbers for references.

The previous phylogenetic analysis used the BVDV-1a reference strain Bega [25]. Otherwise, it clustered into BVDV-1c based on the 5’ UTR and NPro region [36] with strain Bega-like, but no NS5B nucleotide sequence of strain Bega can be found in GenBank [37,38]. Here, strain Bega-like from Australia included five BVDV-1 field samples (2013 and 2015) clustering into BVDV-1c, and their nucleotide sequence identities were 92-95%. Those samples originated from Central Java consisting of Cilacap (MK411751 and MK411763), Banyumas (MK411753), and Semarang (MK411758), and Pasuruan in East Java (MK411752). The mean distance of all five samples and reference within the BVDV-1c group is the smallest between other groups, which was 0.067. BVDV-1c is the major subgenotype of BVDV found in Australia [33,39]. There is a connection between the endemic viruses in an area over a longer period and recently introduced viruses, like by importing animals or frozen semen.

Another subgenotype identified in this study was BVDV-1b. Only one field sample from Banyumas (MK 411760) has a close relationship (93%) with the reference sequence (KP755846), and the average distance of all references in the group was 0.079. The most common circulating BVDV-1 in this study was BVDV-1a subgenotype (60%). The BVDV-1a subgenotype illustrated in the top branch of the phylogenetic tree was originally from Central Java and consists of Banyumas (MK411754 to MK411757, MK411762, MK411764, MK411765), and Boyolali (MK411761) and East Java namely Malang (MK411759) collected from 2013 to 2016. The nine BVDV-1a field samples have a sequence similarity up to 90-93% compared to reference sequence KX170701, and the average distance in the group was 0.071. We found that six BVDV-1a field samples which were from Banyumas (2014 to 2016) and from Malang (2015) had 99-100% of sequence similarity. Three seropositive samples clustered with BVDV-1a, and two samples (MK411764 and MK411765) were absolutely identical (100%). This similarity was assumed to be related to both virus transmission and movement of the infected animal.

This study also showed that BVDV-1a infection in Banyumas (MK411755) had evolved from 2013 to 2016, with only 91-92% of sequence identity. The development of BVDV subgenotypes is caused by an RNA virus that is easily mutated resulting in modification of gene expression that is useful for replication and survival [29]. Mutation often occurred during RNA virus replication that was affected by the RdRp enzyme. The differences in the nucleotide sequence were believed to be the criteria that differentiated BVDV [40]. The fifteen sequenced samples showed three subgenotypes as their genetic diversity, and the seronegative samples had more diversity than seropositive of p80 BVDV.

All BVDV-1 field samples from Central Java and East Java indicated genetic variability and changing amino acids in the NS5B protein. The amino acid sequences from 189-308 were encoded by the 360 bp NS5B nucleotide sequence. The fifteen BVDV-1 field samples had the same R283K point mutation as the three BVDV-1 references from GenBank (Australia, Canada, and USA) compared to BVDV-1 Singer. In the F-motif sequence, the K263, R267, K282, R283, R285, and I287 residues and the last three residues were highly conserved. This is a typical characteristic of the ribonucleotide triphosphate-binding channel [17]. The similarity of this point mutation tends to have a similar characteristic as RdRp enzyme activity.

Changing the amino acid arginine 283 to lysine (R283K) in the sequence motif influenced the activity of the RdRp enzyme. Lysine and arginine are in the same group of positive polar amino acids, so the mutation had an insignificant impact on the activity of the enzyme. Even though changing arginine 283 to lysine (R283K) caused a decrease in RdRp activity and catalysis, its activity and catalysis were still higher than changing R283A. It may be due to ribonucleotide binding and positioning of the template base in the active site. The low activity of the RdRp enzyme was influenced by the decrease in ribonucleotide bond strength and efficiency of catalysis both in the primer-dependent initiation and de novo initiation [19].

The characteristics of the BVDV from 2013 to 2017 were studied based on the NS5B gene, which was dynamic since 2013, with an average pairwise distance of 0.207. The NS5B gene encoded RdRp and was responsible for the transcription and the replication of the genome virus [19,41,42]. The nonstructural NS5B protein of BVDV was a key enzyme in virus replication with an internal sequence motif that could predict the activity of RdRp enzyme [17]. The changing of the amino acid responsible for RdRp enzyme activity influenced the precision of replication and genetic variability in the virus.

## Conclusion

The analysis of data presented in this paper revealed the genetic variability of BVDV from infected cattle in Central Java and East Java, Indonesia. Three subgenotypes of BVDV-1 were identified--BVDV-1a, -1b, and -1c from fifteen field samples based on the NS5B gene. Point mutation R283K in the amino residues of R283, R285, and I287 among all of the sequenced samples were revealed in the nucleotide-binding pocket, and they might impact RdRp enzyme activity involved in viral replication.

## Authors’ Contributions

SHI conceived, arranged, designed, and analyzed the study. SHI. HW, RW, HWi, FSTR, and BP supervised the experiments and corrected the manuscript. All authors read and approved the final manuscript.
